# Salty Biscuits Enriched with Fresh and Dried Bee Pollen: Chemical, Technological, and Sensory Characterization

**DOI:** 10.3390/foods14030527

**Published:** 2025-02-06

**Authors:** Alessandro Bianchi, Sonia Capparelli, Isabella Taglieri, Chiara Sanmartin, Laura Pistelli, Francesca Venturi

**Affiliations:** 1 Department of Agriculture, Food and Environment, University of Pisa, Via del Borghetto 80, 56124 Pisa, Italy; alessandro.bianchi@phd.unipi.it (A.B.); sonia.capparelli@phd.unipi.it (S.C.); isabella.taglieri@unipi.it (I.T.); laura.pistelli@unipi.it (L.P.); francesca.venturi@unipi.it (F.V.); 2 Interdepartmental Research Centre “Nutraceuticals and Food for Health”, University of Pisa, Via del Borghetto 80, 56124 Pisa, Italy; 3 Interdepartmental Center of Agro-Environmental Research “Enrico Avanzi”, University of Pisa, 56122 Pisa, Italy

**Keywords:** fortification, antioxidant activity, carotenoids, functional foods, color, sensory quality, physicochemical properties, bakery products

## Abstract

Bee pollen is a potential functional food ingredient as it contains essential nutrients and a wide range of bioactive compounds. Among bakery products, sweet or salty biscuits are very popular, because they can be consumed quickly, have a long shelf life, and have a favorable taste and texture. The aim of this study was to investigate the effects of the enrichment of salty biscuits with bee pollen (fresh and dried) through their chemical-technological and sensory characteristics. The biscuit formulations were created by replacing the flour with an increasing amount (5% and 10%) of fresh (FP) and dried (DP) pollen. A formulation without pollen was used as the control (CB). To evaluate its potential as a fortification ingredient, pollen as well as salty biscuits were analyzed in terms of their chemical composition and sensory characteristics. In particular, biscuits with 5% fresh pollen (FPB5%) proved to be the formulation with the optimal combination of chemical-compositional and sensory characteristics. Given the increase in their antioxidant component, fortified biscuits can represent an interesting vehicle for phenolic compounds and carotenoids, with a characteristic sensory profile.

## 1. Introduction

In the last few decades, the interest in food enriched with one or more micronutrients and/or nutrients has increased regardless of whether these are usually contained in the food [1,2,3]. In 2006, the constant growth of fortified foods led the European Union to issue EC regulation no. 1924/2006, which regulates the addition of vitamins and minerals to foods, reporting a list of those allowed to go together with their relative forms [2,4]. Many food ingredients (food additives or functional food additives) have been included in the formulation of baked goods to increase their diversity and quality, in terms of nutritional and sensory characteristics [5,6,7].

Sweet or salty biscuits are very popular bakery products, mainly because they can be consumed quickly, have a long shelf life, and have varied tastes and textures [8]. The essential ingredients are flour, oil, sugar and/or salt, water, and chemical leavening agents such as sodium bicarbonate. Regular consumption of high doses of certain ingredients (such as salt, sugar, and fat) may be considered unsafe, particularly when associated with highly processed foods [9,10]. So, in recent years, numerous studies have been conducted to improve the nutritional quality of such products [3,6,11].

Due to its nutritional properties, bee pollen is suitable for food supplementation because, in addition to being rich in nutrients, it contains important bioactive molecules, antioxidants, minerals, and vitamins [12,13,14,15]. These compounds can have beneficial effects on human health, preventing the onset of certain diseases [12,16]. Bee pollen contains important biologically active molecules, such as phenolic acid derivatives and flavonoid glycosides like quercetin and rutin [17,18,19,20]. Flavonoids are secondary plant metabolites known for their anti-inflammatory, anticancer, antiviral, antibacterial, and cardioprotective properties [21,22]. In addition, a fairly significant amount of vitamin B12, a nutrient not typically derived from plant sources, can be found in pollen [15,23,24], which is valuable particularly for vegetarian or gluten-free diets [16,25]. Moreover, bee pollen contains phytosterols, with beta-sitosterol contributing to its anti-teratogenic, anti-inflammatory, and immunostimulant effects [13,18,26].

Several studies [27,28,29,30,31,32,33,34] have demonstrated that adding bee pollen can improve the sensory properties of bread and sweet biscuits, particularly by improving their taste and rheological properties, which influence people’s tactile sensations when chewing [34,35].

The unique techno-functional properties of bee pollen, such as hardness, chewiness, adhesiveness, gumminess, springiness, resilience, and cohesiveness [36], make it an excellent ingredient for incorporation into a wide range of food products [13,37]. Higher concentrations of bee pollen result in softer biscuits and bread [28,37,38]. Additionally, bee pollen’s excellent emulsifying properties make it suitable for use as a natural emulsifier for the development of gluten-free bread [27,39].

A study [28] investigating the enrichment of gluten-free bread with multifloral bee pollen, in increasing percentages, demonstrated that this integration provided a well-leavened dough system. After baking, the enriched bread showed a significant improvement in rheological, technological-functional, and sensory properties compared to the control bread.

Another study [37] compared sweet biscuits enriched with monofloral bee pollen, such as rapeseed pollen (*Brassica napus* L.), phacelia (*Phacelia tanacetifolia* Benth.), and sunflower (*Helianthus annuus* L.) on the basis of their nutritional, physical, and sensory properties. The results confirmed that pollen can be used to improve the nutritional value of biscuits; however, it should be noted that the chemical composition of bee pollen, which varies according to environmental conditions, the plant species from which it is collected, and commercial processing and preservation techniques [14,40,41,42], greatly affects the overall acceptance of the final product [43]. The fortification of biscuits with bee pollen resulted in a statistically significant increase in the sugar, protein, ash, fiber, and polyphenol content, as well as the antioxidant potential of the final product [30,44]. In addition to the nutritional profile, the pollen positively affected the sensory profile of the biscuits, as they were characterized by a greater consistency and a darker surface than the control [30,44].

Sokmen et al. [45] showed that the fortification with multifloral bee pollen on reduced-fat biscuits significantly increased the overall sensory acceptability of the biscuits, their nutritional value, given the reduction in fat, as well as their total phenol content and antioxidant capacity values.

All the studies in the literature report that bee pollen is a natural ingredient that is well suited for fortifying foods such as baked goods [46] or yoghurt [47]. Not only does it enhance these products by providing antioxidant compounds with health benefits, but it also improves their sensory qualities, adding unique flavors, taste, and colors [37,48,49].

Nonetheless, further research is needed to understand the interactions between phenolic compounds and macronutrients in pollen-enriched bakery products and to improve the bio-accessibility of nutrients in these products [18,30,50].

However, to date, no published studies have investigated the application of bee pollen as a fortifying agent in salty biscuits or its effects on their technological-functional and sensory properties.

For this reason, this study aimed to address this gap by producing salty biscuits formulated with different levels (0%, 5%, and 10%) of fresh and dried multifloral bee pollen and evaluating their technological properties, physicochemical composition, and sensory profile both soon after cooking and over time (a timelapse of 60 days).

## 2. Materials and Methods

### 2.1. Raw Materials

The basic ingredients used to produce biscuits were purchased at a local supermarket (Coop Italia, Pisa, Italy), while the bee pollen used was donated by the Mancini agricultural company (Tirli, Grosseto, Italy) and subsequently frozen at −18 °C until further analysis.

The ingredients used for the biscuits’ formulation were soft wheat flour type 00 (Coop Italia, Bologna, Italy), dry white wine (Tavernello, Caviro soc. coop. agricola, Faenza, Ravenna, Italy), extra virgin olive oil (Filippo Berio, Salov S.p.A, Lucca, Italy), sea salt (Coop Italia, Bologna, Italy), still water (Coop Italia, Bologna, Italy), and multifloral bee pollen.

### 2.2. Pollen-Drying Process

A total of 50 g of fresh pollen was dried at a temperature of 35 °C for 24 h in the Fruit Jerky Plus 6 dryer (Klarstein, Berlin, Germany) at the Department of Agricultural, Food, and Environment of the University of Pisa until reaching approximately 12% humidity.

### 2.3. Process of Making Salty Biscuits 

The biscuit samples were prepared at room temperature (23 ± 2 °C) by replacing wheat flour with fresh (FP) or dried (DP) bee pollen, in the amounts of 5% and 10%. A control (CB) sample (pollen-free biscuits, 0%) was also prepared. To reduce bias, in samples prepared with DP, the needed amount of water was added to reach the same final fresh weight of 294 g.

The formulations used were reported in Table 1 and the samples were coded as follows:

- CB: control salty biscuits without pollen;
- FPB5%: salty biscuits with 5% fresh pollen;
- FPB10%: salty biscuits with 10% fresh pollen;
- DPB5%: salty biscuits with 5% dried pollen;
- DPB10%: salty biscuits with 10% dried pollen.

All the ingredients were weighed in separate containers. The flour, salt, and olive oil were mixed until a crumbly mixture was obtained. The pollen was dissolved in the wine and added to the mixture. The dough was kneaded by hand until a homogeneous consistency was obtained. The dough was then shaped into biscuits approximately 3 × 3 × 0.5 cm in size.

The biscuits were baked in an electric convection oven (Fimar, OMNIA srl, Santarcangelo di Romagna, Rimini, Italy) at 160 °C for 40 min, subsequently cooled to room temperature, packaged in plastic bags (with an outer nylon layer and two plastic layers; Food Saver, Moncalieri, Torino, Italy) with an industrial packaging machine (450 GAS Lavezzini, Fiorenzuola d’Arda, Piacenza, Italy), and stored at 23 ± 2 °C. The entire biscuit-making process was repeated in triplicate, and three different batches were obtained for each formulation.

### 2.4. Technological and Chemical Composition of Bee Pollen and Biscuits

The dry matter (dm) was determined for a sample of approximately 5 g (bee pollen and biscuits) dried at 105 °C until constant weight was achieved, and the pH of biscuits was measured with a pH meter (pH 80+ DHS, XS Instrument, Modena, Italy) according to the AACC (American Association of Cereal Chemists) standard method, as previously reported [3].

Total titratable acidity (TTA) was determined in biscuits following Bianchi et al.’s study [51].

Water activity (a_w_) of bee pollen and biscuits was measured by a HygroPalm HP23-AW-A equipment (Rotronic AG, Bassersdorf, Switzerland) [52].

Free acidity (FA) (% oleic acid) was determined in biscuits as previously reported [53]; 4 g of ground biscuits was added to 100 mL of 50% ethanol and stirred for 3 h at 22 °C. The solution was filtered, and 50 mL was then titrated with NaOH (0.02 N) until the indicator (phenolphthalein) changed.

Peroxide value (meq O_2_/kg dm) was determined in biscuits as reported by Nanditha et al., 2009 [50].

For the evaluation of phytochemical compounds (total polyphenols, flavonoids, and antioxidant activity), 1 g of pollen (fresh and dried) and 5 g of biscuit sample were extracted at room temperature using 20 mL of a methanol/water solution (80% *v*/*v*) and sonicated for 30 min at a frequency of 2400 Mhz. The samples were centrifuged at 10,000× *g* rpm for 10 min at 23 °C, and the supernatants were collected and used for further analyses involving a spectrophotometer assay.

Total polyphenols were determined at 765 nm using the Folin–Ciocalteau method [54]. The results are expressed as mg gallic acid equivalent (GAE) per g dm.

Flavonoids were determined as reported by Bianchi et al. [55]. The absorbance was read at 510 nm, while the concentration of the samples was expressed as mg of catechin equivalents (CE) per g dm, comparing the measures to a standard curve of catechin.

Antioxidant activity was measured by three methods [56]: ABTS (2,2′-azino-bis [3-ethylbenzothiazoline-6-sulfonic acid]) by reading the absorbance at 734 nm, DPPH (2,2-diphenyl-1-picrylhydrazyl) by reading the absorbance at 515 nm, and FRAP (ferric ion reducing antioxidant power) by reading the absorbance at 593 nm. The results were expressed as µmol Trolox equivalents (TE) per g dm of sample, based on different standard curves of Trolox: 0–200 µmol L^−1^ for the DPPH assay, 0.2–1.5 mM range for ABTS, and 0–2.0 mM for the FRAP assay.

For carotenoid determination in bee pollen and biscuits, extraction (with saturated n-butanol) was performed following the methodology of Cabras [53], and concentration was determined at 435.8 nm expressed as mg of *β*-carotene per kg dm of biscuits or bee pollen, comparing the measurements to a standard curve of *β*-carotene.

All tests were performed in triplicate.

### 2.5. Colorimetric Analysis of Bee Pollen and Biscuits

The color of the bee pollen and biscuits was measured according to the CIE L*a*b* Color System using a tristimulus colorimeter (CLM-196 Benchtop, Eoptis, Trento, Italy). The analysis was performed on pollen and biscuit samples placed on an area of approximately 4.5 cm^2^, and each sample was analyzed in triplicate.

The color was defined based on the chromatic coordinates, lightness (L*), green-red (a*), and blue-yellow components (b*). The cylindric coordinates, chroma (C*) and hue (h*), were also calculated as previously reported [57]. The color differences among samples (∆E^*^_ab_) were calculated as previously reported [3] and are expressed in CIELAB units.

The whiteness (WI) and yellowness (YI) indices of the samples were calculated according to the equations provided by Bianchi et al. [3].

### 2.6. Sensory Evaluation of Biscuits

Sensory profiles of the biscuits were determined through quantitative descriptive analysis (QDA) from the “expert panel” of the University of Pisa.

QDA was conducted at room temperature, in a standard sensory laboratory (ISO 8589:2010 [58]) located in the Department of Agriculture, Food, and Environment (DAFE) by trained judges (10 assessors: 6 females and 4 males, aged between 23 and 63 years), who were primarily experts in bakery tasting. The assessors were selected based on their availability from a larger pool of judges who regularly collaborate with the DAFE at the University of Pisa. All judges underwent standardized training to enhance their ability to recognize, describe, and quantify tastes, odors, and texture properties in accordance with ISO 8586:2023 standard [59] and typically worked collaboratively.

Assessor records, including consent forms and personal data, were collected and maintained in compliance with national data protection laws. Sensory activities were conducted in consideration of ethical principles regarding the use of human subjects and adhered to health and safety regulations. The research obtained the approval of the bioethical committee of the University of Pisa (protocol n. 0088081/2023).

Prior to the tasting sessions of the experimental samples, a sub-group of the trained panelists participated in a consensus panel specifically designed to generate descriptors and their definitions.

A final set of 27 descriptive parameters was established through consensus among the panelists for the QDA sensory test. These included both quantitative descriptors (such as color intensity, hue (yellow-ochre), shape regularity, olfactory intensity, persistence, and specific sensory attributes like oily, grainy, floral, fruity, vegetal, frankness, toasted, chewing resistance, cohesiveness, hardness, friability, sweetness, saltiness, acidity, bitterness, astringency, umami, and aftertaste) and hedonic descriptors (such as visual attractiveness, olfactory pleasantness, taste pleasantness, and overall pleasantness). To facilitate the evaluation, an innovative sensory sheet specifically designed for tasting bee pollen biscuits was created (Figure S1).

The samples were presented in a different order at each tasting session, and 10 min intervals between each sample were set. Furthermore, a biscuit sample was randomly replicated to verify the performance of the panel at each tasting session. For evaluation, each assessor was provided with filtered water and asked to cleanse their palate between tastings.

Each attribute was evaluated on a 0–9 scale. All ratings were digitally acquired by the Input Sensory Soft 2.0 (ISS, Centro Studi Assaggiatori, Brescia, Italy). Finally, the overall hedonic index (HI) of the biscuits, which represents the overall acceptability of the product, was calculated based on the mean of the hedonic parameters, which was converted to a scale from 0 to 10, as previously reported [60]. The HI was calculated at time 0 (after cooling) and after 30 and 60 days of storage at 23 ± 2 °C in plastic bags to assess whether the acceptability of the product changed over time.

### 2.7. Statistical Analysis

Results were statistically analyzed by one-way analysis of variance (ANOVA) by the software CoStat 6.451 (CoHort Software, Pacific Grove, CA, USA), using Tukey’s honestly significant difference test (*p* < 0.05).

The data processing of the sensory profile was carried out using the software Big Sensory Soft 2.0 (Centro Studi Assaggiatori, Brescia, Italy), and the statistical analyses were performed by interquartile two-way ANOVA, choosing samples and panelists as main factors. The correlation value between the quantitative descriptor and the hedonic one was calculated using Pearson correlation coefficient.

Finally, the graphic was created using the software JMP Pro 17 (SAS Institute, Cary, NC, USA).

## 3. Results and Discussion

### 3.1. Characterization of Dried and Fresh Bee Pollen

The physicochemical and technological characterization of fresh (FP) and dried (DP) bee pollen is reported in Table 2.

As expected, following the drying process, the water activity and flavonoid content decrease significantly. In contrast, the dry matter and carotenoids significantly increased in the dry pollen (DP). The total polyphenols and antiradical activity did not show significant variation during the drying process.

No significant differences were observed in the chromatic coordinates and colorimetric parameters between the fresh and dried pollen samples (Table 2). Both fresh and dried pollen grains have a color close to red (+a) and yellow (+b), characterized by a strong color intensity, as indicated by the colorimetric parameter (C*). The yellow index (YI) was higher than the white index (WI). As expected, the color difference (ΔE*_ab_) was found to be two, indicating no perceptible difference to the human eye [61].

### 3.2. Characterization of Biscuits

#### 3.2.1. Chemical-Technological Characterization

The biscuits were analyzed by evaluating the following chemical-technological parameters, which provide information for defining the shelf life of the product (Table 3).

The water activity does not seem to be affected by fortification; the values are close to the range defined as optimal for this type of baked product (a_w_ = 0.30) [62]. As observed, 5% and 10% pollen fortification, as well as pollen drying, did not have any significant effect on the parameters of titratable acidity (TTA), dry matter, pH, and peroxide values (Table 3).

As expected, however, the water activity was influenced by the drying process of the pollen, with a decrease in value (Table 3).

The peroxide value (PV) falls within the legal limits, which for dry biscuits is less than 10 meq O_2_/kg dm [63]. This suggests the good oxidative stability of the salty biscuits.

In contrast, there is a significant variation in the value of the free acidity (FA) of the biscuits likely due to the lipids present in pollen [12,64,65]. However, these values are low and should not affect the oxidative stability of the product.

#### 3.2.2. Phytochemical Characterization

As expected, the addition of fresh or dried pollen resulted in notable differences in the phytochemical components compared to the control sample (Table 4).

In particular, the content of total phenolic compounds increased significantly in the enriched biscuit, compared to the control sample, in line with the 5–10% fortification. The increase is particularly marked for polyphenols, ranging from the value of 0.67 mg GAE/g dm in the control sample (CB) to 2.77 mg GAE/g dm in FPB10% and to 3.06 mg GAE/g in BPB10% (Table 4). These results fall within the range previously reported by other authors [29,45].

Flavonoids are also affected by this increase and the differences are statistically significant (*p* < 0.01). This increase is more pronounced in biscuits with fresh pollen (FP) compared to those with dried pollen (DP).

Similarly, the results of the antioxidant capacity (ABTS, DPPH, and FRAP) showed an increasing trend together with the percentage of fresh or dried pollen added to the product [45]. The increase is particularly marked for DPPH and FRAP, with values rising from 0.94 μmol TE/g dm and 1.71 μmol TE/g dm, respectively, in CB to 7.29 μmol TE/g and 9.05 μmol TE/g (FBP10%) and 8.75 μmol TE/g and 15.54 μmol TE/g (DBP10%) (Table 4).

Regarding carotenoids, the content significantly increased as the quantity of pollen increased, going from 3.69 mg *β*-carotene/kg dm (CB) to 11.36 mg *β*-carotene/kg dm (FBP5%) and 26.48 mg *β*-carotene/kg dm (FBP10%) for the biscuits with fresh pollen and from 12.50 mg *β*-carotene/kg dm (DBP5%) to 15.69 mg *β*-carotene/kg dm (DBP10%) for those enriched with dried pollen. It is also possible to assert that with the same percentage of fortification, following cooking, the content of these molecules increases [45,66]. The increase could be explained both by the hydrolysis of bound molecules and the presence of fats (e.g., oil) in the dough, which protects carotenoids, improves their stability, and limits oxidation. Proteins and phenolic compounds in pollen can further stabilize carotenoids during cooking.

#### 3.2.3. Color Characterization

The addition of pollen made the biscuits different from a chromatic point of view. Analyzing the color of the biscuits, it was observed that the addition of fresh or dried pollen significantly darkened the surface of the biscuits compared to the control (Table 5).

Since pollen is darker than wheat flour, the brightness (L*) of the 5% and 10% fortified biscuits was significantly lower than the CB; it is hypothesized that the products of the Maillard reaction increase with the increase in the concentration of pollen due to the high amount of amino acids [31,67].

As the quantity of pollen added increased, a significant increase in the red (+a) and yellow (+b) (Table 5) in the biscuits was observed, presumably associated with the main pollen pigments, such as carotenoids and flavonoids [19,24]. Furthermore, the results related to the chromatic value (C*) suggest that the fortification with pollen significantly increases the color intensity of the biscuits, compared to the control. As expected, there was a decrease in the white index (WI) and an increase in the yellow index (YI), respectively (Table 5).

Furthermore, the color differences (ΔE*_ab_) among the samples were calculated (Table 6).

The results indicate that the 5% (FPB5% and DPB5%) and 10% (FPB10% and DPB10%) fortified biscuits have very different colors compared to the control (ΔE*_ab_ > 12).

There was also a difference in color between the samples with 5% and 10% fortification (Figure S2), but it was significantly higher in the samples with dried pollen (6 < ΔE*_ab_ < 12) compared to the fresh ones (3 < ΔE*_ab_ < 6). The color was a fundamental parameter that significantly influenced the consumers’ perceptions and decisions. A uniform golden color was often associated with well-baked and appetizing biscuits. If the color was too dark, it may have suggested that the biscuits were burnt or had a bitter taste [68].

#### 3.2.4. Sensory Characterization

**Figure 1:**
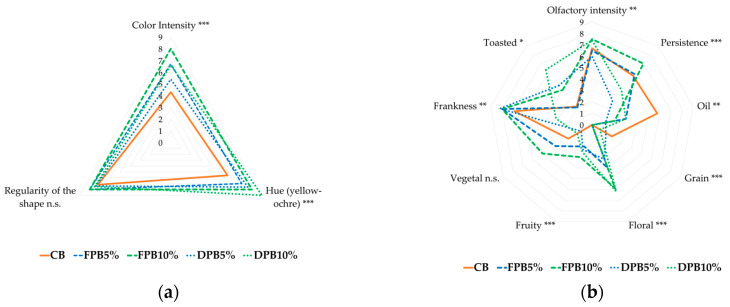
Sensory evaluation: (**a**); Visual descriptors; (**b**) Olfactory descriptors. Significance level: *** = *p* < 0.001; ** = *p* < 0.01; * = *p* < 0.05; n.s. = not significant (*p ≥* 0.05). CB: control salty biscuits without pollen; FPB5%: salty biscuits with 5% fresh pollen; FPB10%: salty biscuits with 10% fresh pollen; DPB5%: salty biscuits with 5% dried pollen; DPB10%: salty biscuits with 10% dried pollen.

From the visual descriptors (Figure 1a), it can be observed that the addition of pollen, fresh and dried, resulted in significant visual differences compared to the control sample, in line with what was expected based on the colorimetric evaluations. In fact, there were evident significant differences in terms of color intensity and hue (from yellow to ochre), which increased as the percentage of pollen increased.

Almost all the olfactory descriptors analyzed (Figure 1b) showed significant differences. Enrichment with pollen was associated with high values of frankness, intensity, and olfactory persistence. The use of pollen determines a higher perception of floral, fruity, and vegetal aromatic notes; the CB sample, on the other hand, is distinguished by a clearer perception of the notes of oil and wheat. Toasted notes also emerge for the DPB10% sample.

**Figure 2:**
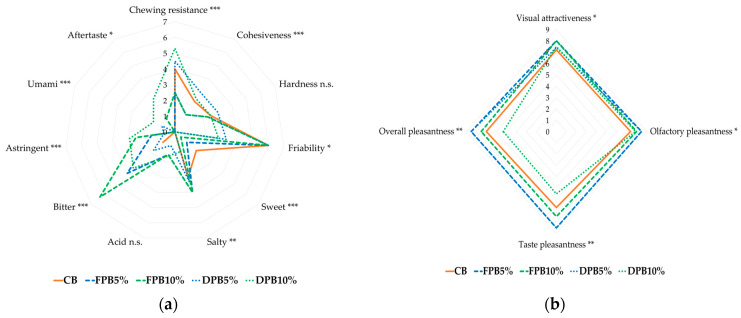
Sensory evaluation: (**a**) Taste descriptors; (**b**) Hedonic descriptor. Significance level: *** = *p* < 0.001; ** = *p* < 0.01; * = *p* < 0.05; n.s. = not significant (*p ≥* 0.05). CB: control salty biscuits without pollen; FPB5%: salty biscuits with 5% fresh pollen; FPB10%: salty biscuits with 10% fresh pollen; DPB5%: salty biscuits with 5% dried pollen; DPB10%: salty biscuits with 10% dried pollen.

From the taste descriptors (Figure 2a), the enrichment with pollen makes the biscuits more astringent, with higher hints of bitterness that resulted in a more complex taste profile, compared to the control sample, which is perceived as sweeter.

As regards the evaluation of rheological properties, the greatest differences are perceived for chewing resistance and cohesiveness. In chewing resistance, the highest scores are shown by fortified biscuits with dried pollen and control samples, and the same is true for cohesiveness. On the other hand, biscuits enriched with fresh pollen are characterized by greater crumbliness. However, the addition of pollen does not seem to significantly affect the hardness of the product.

The hedonic descriptors (visual attractiveness, olfactory pleasantness, taste pleasant-ness, and overall pleasantness) were all significant (Figure 2b), especially in regards to taste and the level of global satisfaction, for which DPB10% showed the lowest value.

In general, the sensory profile of the fortified biscuits appears to be much broader and they received higher scores for positive taste and smell descriptors than the control, which was the least aromatically favorable.

In addition, it is essential to be aware of which descriptors contribute most to the characterization of the samples. Figure S3 shows the weight assumed by each quantitative and hedonic descriptor on a scale from zero (minimum) to one (maximum).

As can be seen, visual descriptors, such as the intensity of color and hue, as well as olfactory descriptors such as the perception of oily, floral, toasted, and frankness notes, contribute to a greater extent to the characterization of biscuits, while taste descriptors include resistance to chewing, crumbliness, and a hint of bitterness. As expected, the hedonic parameters (visual attractiveness, olfactory pleasantness, gustatory pleasantness, and overall pleasantness) contribute equally to the characterization of the product.

**Figure 3:**
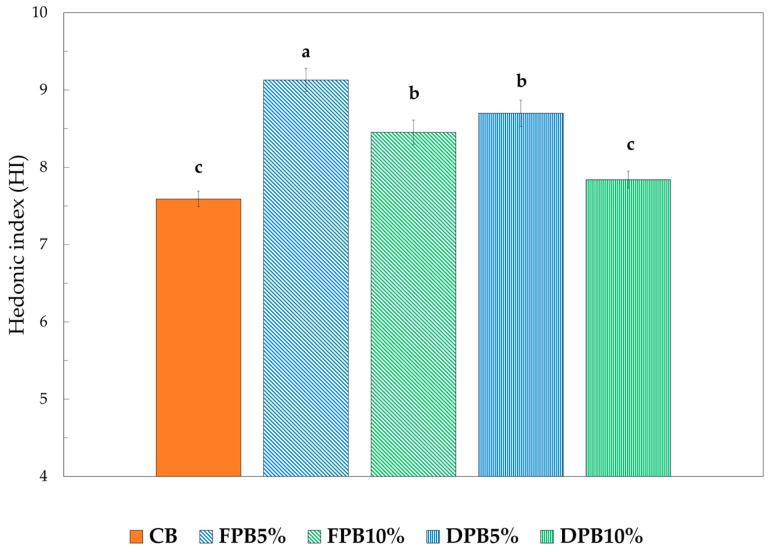
Hedonic index at time 0. Different letters indicate significant difference among values (*p* < 0.05). CB: control salty biscuits without pollen; FPB5%: salty biscuits with 5% fresh pollen; FPB10%: salty biscuits with 10% fresh pollen; DPB5%: salty biscuits with 5% dried pollen; DPB10%: salty biscuits with 10% dried pollen.

Figure 3 shows the HI calculated for the biscuits analyzed. The hedonic quality expressed by a product represents a fundamental value determining its success. 

It can be seen that the FPB5% group obtained the highest hedonic index. In general, it can be seen that the formulations with fresh pollen showed higher values than the biscuits with pollen dried at the same percentage, as well as control samples. However, all formulations showed overall hedonic indices greater than the acceptability index (equal to six) [51,60].

**Figure 4:**
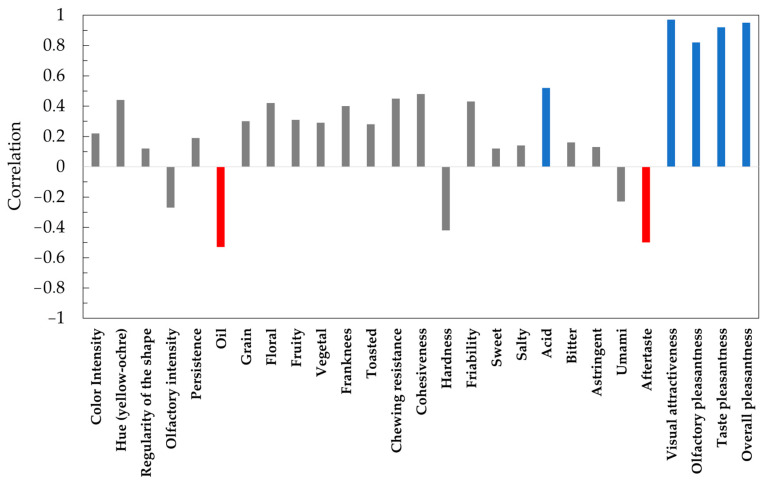
Correlation value for the quantitative and hedonic descriptors. Red color: significant negative effect; Blue color: significant positive effect; Grey color: no significant effect.

While the acceptability of a product is important, what is even more critical is identifying which features to highlight or downplay in order to improve the product. This enables targeted product innovation, optimizing the use of resources for maximum effectiveness. The hedonic generators in Figure 4 show to what extent the descriptor contributed to the pleasantness highlighted by the panel.

Characters that negatively affect hedonic quality are highlighted in red, and those that increase hedonic quality are highlighted in blue. For the statistical technique used, values over 0.30, both positive and negative, must be considered; over −0.5 and +0.5, the correlation is significant.

The most consistent contribution to the hedonic index is due to the hedonic descriptors themselves: taste pleasantness (+0.97), olfactory pleasantness (+0.92), global (+0.95), and visual attractiveness (+0.82), followed by the acidity (+0.52), which is probably correlated with the sensory perception of the pollen itself. Oil (−0.53) and aftertaste (−0.51), on the other hand, negatively affect the hedonic quality of the product. Other descriptors that, albeit to a lesser extent, have contributed positively to the hedonic quality of the product were frankness (the absence of olfactory defects), fruity and floral aromatic notes, hue, and friability.

Shelf-life refers to the length of time between the production of a food and the loss of those characteristics that make it acceptable to the consumer [69]. In the case of dry biscuits, the limit used to determine this acceptability is often based on the level of sensory quality [60].

**Figure 5:**
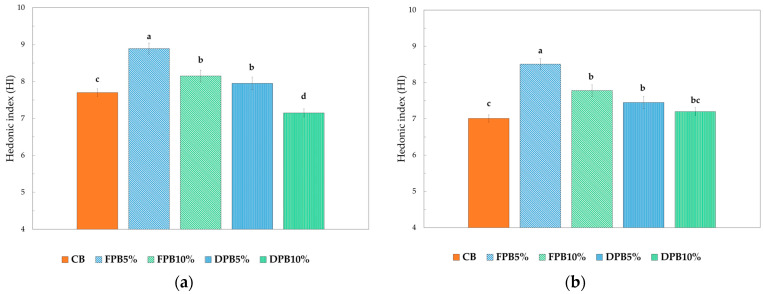
Hedonic index (HI): (**a**) After 30 days of storage; (**b**) After 60 days of storage. Different letters indicate significant difference among values (*p* < 0.05). CB: control salty biscuits without pollen; FPB5%: salty biscuits with 5% fresh pollen; FPB10%: salty biscuits with 10% fresh pollen; DPB5%: salty biscuits with 5% dried pollen; DPB10%: salty biscuits with 10% dried pollen.

During the present study, sensory quality was therefore evaluated after 30 and 60 days of storage at a temperature of 23 ± 2 °C inside a plastic film bag. The data collected were synthesized through the hedonic index to evaluate their changes over time (Figure 5a,b). It can be observed that in the period of time considered, the fortified biscuits, both with fresh and dried pollen, have consistently maintained their sensory properties, and none of the samples evaluated received an overall evaluation below the limit of acceptability.

## 4. Conclusions

The process of the fortification of the biscuits with bee pollen enabled us to obtain a high-quality finished product with an improved nutritional and sensory profile, enriched in bioactive compounds that increase its nutraceutical profile.

Based on the statistical analysis, adding 10% bee pollen appears to be the most effective for enriching biscuits with phenolic and carotenoid compounds, thereby enhancing their antioxidant activity. This supplementation level provides maximum nutritional benefits. However, even a 5% addition of pollen significantly improved these chemical and compositional parameters. Overall, biscuits fortified with 5% fresh pollen emerged as the optimal formulation, balancing chemical, technological, and sensory properties, including distinctive flavors and colors. This suggests that fortifying biscuits with pollen could greatly expand the availability of health-promoting snacks and broaden the potential applications of bee pollen in the food industry.

Furthermore, based on the data obtained, it could be suggested that the choice between fresh or dried pollen did not significantly affect the analyzed parameters of the biscuits. This could be explained by the mild drying treatment, applied to the pollen, which resulted in a final moisture content of 12%, above the established commercial limits of 2–4%.

Finally, it was observed that, despite the short observation time, the fortified biscuits maintained their sensory properties consistently over time.

## 5. Future Prospects

As a future goal, it would be valuable to implement a more targeted drying treatment for pollen to better understand its impact on fortified biscuits and, ultimately, to extend this approach to gluten-free products. In addition, conducting stability tests on the enriched biscuits, integrated with the sensory evaluation and chemical characterization of the samples together with microbiological analyses over time, could help determine their shelf-life. This approach would enable the development of a product that combines chemical, sensory, and nutritional qualities and a long shelf-life.

## Figures and Tables

**Table 1:** Formulations adopted to produce about 300 g of salty biscuit dough.

Ingredients (g)	CB	FPB5%	FPB10%	DPB5%	DPB10%
Soft wheat flour type 00	184	174.8	165.6	174.8	165.6
Water	0	0	0	1.0	2.0
Multifloral bee pollen	0	9.2	18.4	8.2	16.4
Dry white wine	67	67	67	67	67
Extra virgin olive oil	40	40	40	40	40
Sea salt	3	3	3	3	3
Total fresh weight	294	294	294	294	294

**Table 2:** Physicochemical and technological characterization of fresh (FP) and dried (DP) pollen.

Parameters	*p*-Value ^1^	FP	DP
Dry matter (%)	***	72.93 ± 0.46 ^b^	88.06 ± 0.12 ^a^
Water activity (a_w_)	***	0.713 ± 0.003 ^a^	0.353 ± 0.002 ^b^
Total polyphenols (mg GAE/g dm)	n.s.	25.97 ± 0.72	25.57 ± 1.15
Flavonoids (mg CE/g dm)	**	10.77 ± 0.30 ^a^	8.45 ± 0.11 ^b^
Carotenoids (mg/kg dm)	***	100.35 ± 0.43 ^b^	117.54 ± 1.95 ^a^
ABTS (μmol TE/g dm)	n.s	227.74 ± 3.41	225.56 ± 4.77
DPPH (μmol TE/g dm)	n.s.	19.03 ± 3.68	25.32 ± 7.11
FRAP (μmol TE/g dm)	n.s.	68.44 ± 0.53	72.44 ± 3.27
L*	n.s.	54.39 ± 2.71	52.68 ± 1.84
a*	n.s.	0.30 ± 0.86	1.24 ± 0.90
b*	n.s.	35.07 ± 4.41	35.65 ± 3.10
C*	n.s.	35.07 ± 4.42	35.68 ± 3.13
h*	n.s.	89.62 ± 1.26	88.06 ± 1.25
White index (WI)	n.s.	42.31 ± 0.80	40.67 ± 0.89
Yellow index (YI)	n.s.	91.89 ± 6.93	96.58 ± 5.49
ΔE*_ab_			2.0

Values are presented as the mean ± standard deviation (SD) of 3 samples. In the same row, different letters indicate significant difference among values. ^1^ Significance level: *** = *p* < 0.001; ** = *p* < 0.01; n.s. = not significant (*p ≥* 0.05). FP: fresh pollen; DP = dried pollen. ΔE*_ab_ was calculated by taking fresh pollen as a reference to evaluate the difference after drying.

**Table 3:** Chemical-technological parameters of different salty biscuits.

Parameters	*p*-Value ^1^	CB	FPB5%	FPB10%	DPB5%	DPB10%
Dry matter (%)	n.s.	96.93 ± 0.59	96.60 ± 0.36	96.02 ± 0.21	96.68 ± 0.95	97.75 ± 0.36
a_w_	***	0.134 ± 0.030 ^c^	0.208 ± 0.003 ^b^	0.274 ± 0.006 ^a^	0.119 ± 0.001 ^c^	0.123 ± 0.001 ^c^
pH	*	5.44 ± 0.01 ^ab^	5.40 ± 0.04 ^b^	5.52 ± 0.04 ^a^	5.43 ± 0.02 ^ab^	5.50 ± 0.01 ^b^
TTA (meq/g dm)	n.s.	13.04 ± 0.37	13.76 ± 1.04	11.88 ± 0.99	12.76 ± 0.49	12.02 ± 0.49
FA (% oleic acid dm)	**	0.06 ± 0.01 ^b^	0.10 ± 0.01 ^a^	0.11 ± 0.01 ^a^	0.09 ± 0.01 ^a^	0.11 ± 0.01 ^a^
PV (meq O_2_/kg dm)	n.s.	2.79 ± 0.06	2.43 ± 0.22	2.86 ± 0.37	2.33 ± 0.37	2.76 ± 0.29

Values are presented as the mean ± standard deviation (SD) of 3 samples. In the same row, different letters indicate significant difference among values. ^1^ Significance level: *** = *p* < 0.001; ** = *p* < 0.01; * = *p* < 0.05; n.s. = not significant (*p* ≥ 0.05). CB: control salty biscuits without pollen; FPB5%: salty biscuits with 5% fresh pollen; FPB10%: salty biscuits with 10% fresh pollen; DPB5%: salty biscuits with 5% dried pollen; DPB10%: salty biscuits with 10% dried pollen.

**Table 4:** Phytochemical parameters of different salty biscuits.

Parameters	*p*-Value ^1^	CB	FPB5%	FPB10%	DPB5%	DPB10%
Total polyphenols (mg GAE/g dm)	***	0.67 ± 0.09 ^d^	1.83 ± 0.14 ^c^	2.77 ± 0.15 ^b^	2.03 ± 0.20 ^c^	3.06 ± 0.07 ^a^
Flavonoids (mg CE/g dm)	**	0.20 ± 0.01 ^d^	0.28 ± 0.01 ^bc^	0.60 ± 0.04 ^a^	0.29 ± 0.04 ^bc^	0.42 ± 0.07 ^b^
Carotenoids (mg β-carotene/kg dm)	***	3.96 ± 0.04 ^e^	11.36 ± 0.64 ^d^	18.96 ± 0.22 ^b^	12.50 ± 0.33 ^c^	20.53 ± 0.30 ^a^
ABTS (μmol TE/g dm)	***	2.44 ± 0.17 ^d^	5.22 ± 0.30 ^c^	7.29 ± 0.16 ^b^	5.92 ± 0.46 ^c^	8.75 ± 0.70 ^a^
DPPH (μmol TE/g dm)	***	0.94 ± 0.21 ^d^	2.29 ± 0.07 ^c^	3.26 ± 0.09 ^b^	3.63 ± 0.24 ^b^	5.06 ± 0.28 ^a^
FRAP(μmol TE/g dm)	***	1.71 ± 0.10 ^d^	6.28 ± 0.16 ^c^	9.05 ± 0.64 ^b^	10.73 ± 0.40 ^b^	15.54 ± 0.43 ^a^

Values are presented as the mean ± standard deviation (SD) of 3 samples. In the same row, different letters indicate significant difference among values. ^1^ Significance level: *** = *p* < 0.001; ** = *p* < 0.01. CB: control salty biscuits without pollen; FPB5%: salty biscuits with 5% fresh pollen; FPB10%: salty biscuits with 10% fresh pollen; DPB5%: salty biscuits with 5% dried pollen; DPB10%: salty biscuits with 10% dried pollen.

**Table 5:** Color parameters of different salty biscuits.

Parameters	*p*-Value ^1^	CB	FPB5%	FPB10%	DPB5%	DPB10%
L*	***	70.70 ± 1.43 ^a^	62.71 ± 1.80 ^b^	57.90 ± 1.03 ^c^	54.63 ± 1.10 ^d^	47.11 ± 0.81 ^e^
a*	***	5.10 ± 1.30 ^d^	6.98 ± 0.75 ^c^	8.02 ± 0.24 ^c^	9.81 ± 0.09 ^b^	11.43 ± 0.36 ^a^
b*	***	31.68 ± 1.15 ^d^	39.49 ± 0.38 ^a^	40.63 ± 0.65 ^a^	36.64 ± 0.06 ^b^	34.68 ± 1.33 ^c^
C*	***	32.11 ± 0.93 ^c^	40.11 ± 0.42 ^a^	41.41 ± 0.65 ^a^	37.93 ± 0.07 ^b^	36.52 ± 1.37 ^b^
h*	***	80.81 ± 2.63 ^a^	79.98 ± 1.05 ^ab^	78.83 ± 0.34 ^b^	75.02 ± 0.13 ^c^	71.75 ± 0.20 ^d^
White index (WI)	***	56.51 ± 0.28 ^a^	45.22 ± 1.34 ^b^	40.94 ± 0.41 ^c^	40.86 ± 0.85 ^c^	35.72 ± 0.44 ^d^
Yellow index (YI)	***	64.02 ± 1.03 ^d^	90.02 ± 2.69 ^c^	100.25 ± 0.80 ^ab^	95.86 ± 1.91 ^b^	105.15 ± 2.74 ^a^

Values are presented as the mean ± standard deviation (SD) of 3 samples. In the same row, different letters indicate significant difference among values. ^1^ Significance level: *** = *p* < 0.001. CB: control salty biscuits without pollen; FPB5%: salty biscuits with 5% fresh pollen; FPB10%: salty biscuits with 10% fresh pollen; DPB5%: salty biscuits with 5% dried pollen; DPB10%: salty biscuits with 10% dried pollen.

**Table 6:** Color differences (ΔE*_ab_) among the different salty biscuits.

ΔE*_ab_	CB	FPB5%	FPB10%	DPB5%	DPB10%
**CB**		11.33	15.88	17.46	24.60
**FPB5%**			5.05	9.02	16.92
**FPB10%**				5.45	12.78
**DPB5%**					7.94
**DPB10%**					

CB: control salty biscuits without pollen; FPB5%: salty biscuits with 5% fresh pollen; FPB10%: salty biscuits with 10% fresh pollen; DPB5%: salty biscuits with 5% dried pollen; DPB10%: salty biscuits with 10% dried pollen.

## Data Availability

The original contributions presented in the study are included in the article/Supplementary Material; further inquiries can be directed to the corresponding author.

## Supplementary Materials

The following supporting information can be downloaded at: https://www.mdpi.com/article/10.3390/foods14030527/s1, Figure S1: Sensory sheet used by the judges; Figure S2: Images of the biscuits after cooking. CB: control salty biscuits without pollen, FPB5%: salty biscuits with 5% fresh pollen, FPB10%: salty biscuits with 10% fresh pollen, DPB5%: salty biscuits with 5% dried pollen, DPB10%: salty biscuits with 10% dried pollen; Figure S3: Final weight assumed by each descriptor.
