# Supplementary material for: Salty Biscuits Enriched with Fresh and Dried Bee Pollen: Chemical, Technological, and Sensory Characterization
Source: Foods. 2025 Feb 6;14(3):527. doi: 10.3390/foods14030527 (PMC11817459; doi:10.3390/foods14030527)
Supplement: Supplementary file 1 [file foods-14-00527-s001.zip › foods-3445241-supplementary.pdf]

Sensory sheet

Name ....

Date ....

**VIEW**	0 5 9
Color Intensity	
Hue (yellow-ochre)	
Regularity of the shape	
Visual attractiveness	

**SMELL**	0 5 9
Olfactory intensity	
Persistence	
Grain	
Floral	
Fruity	
Vegetal	
Frankness	
Toasted	
Oil	
Olfactory pleasantness	

**TASTE**	0 5 9
Chewing resistance	
Cohesiveness	
Hardness	
Friability	
Sweet	
Salty	
Acid	
Bitter	
Astringent	
Umami	
Aftertaste	
Taste pleasantness	

	0 5 9
Overall pleasantness	

Figure S1. Sensory sheet used by the judges.

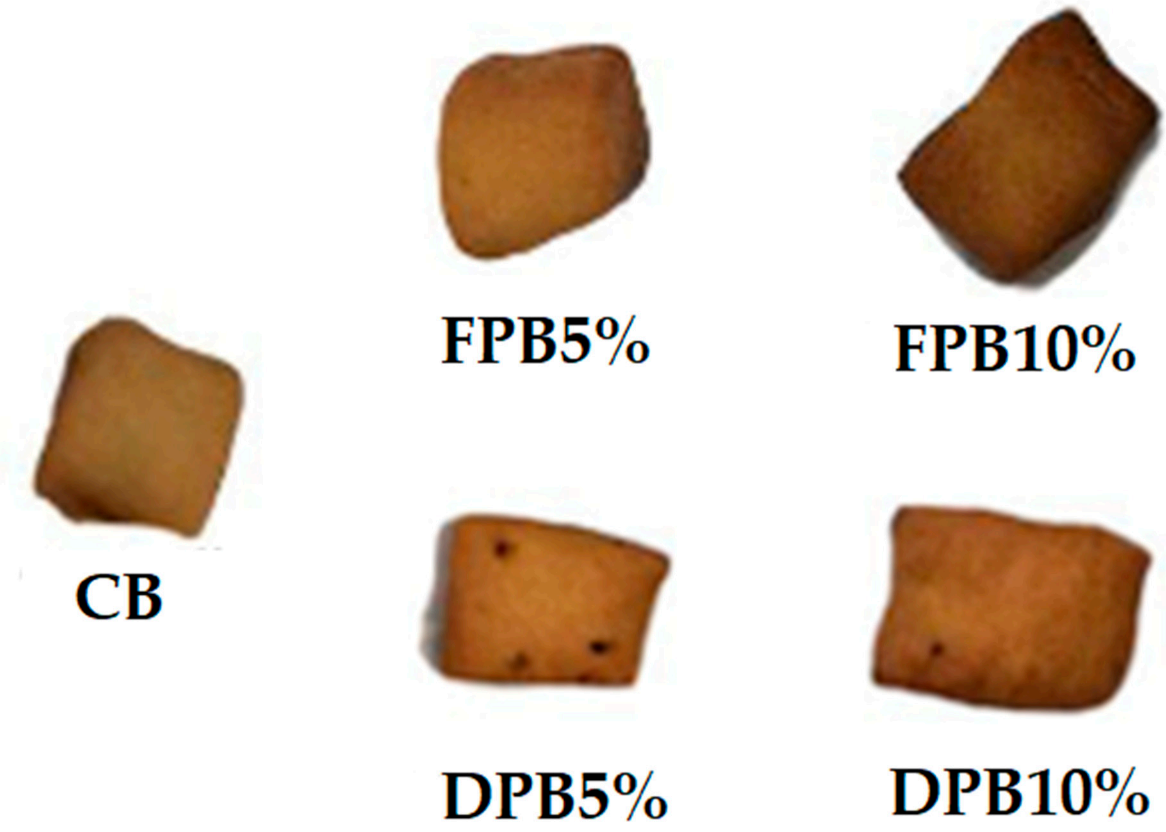

Figure S2. Images of the biscuits after cooking.

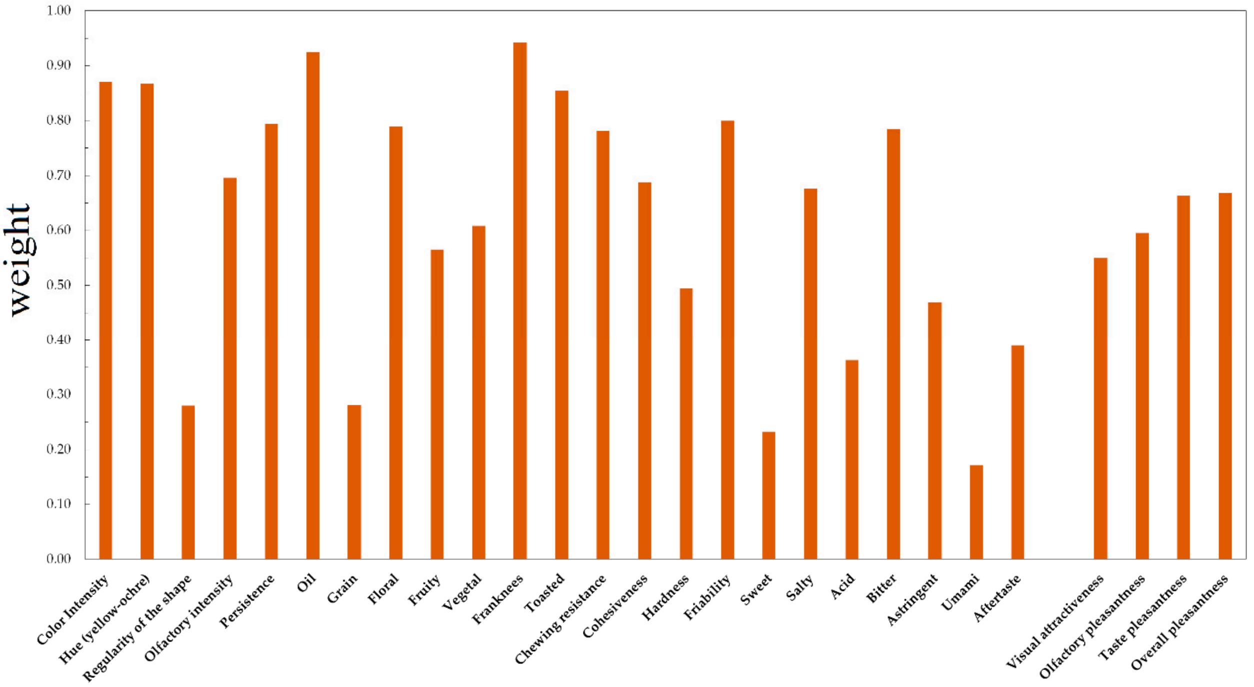

Figure S3. Final weight assumed by each descriptors.
